# Severity of structural lumbar spinal stenosis does not impact responsiveness to exercise-based rehabilitation

**DOI:** 10.1093/ptj/pzag084

**Published:** 2026-08-08

**Authors:** Bahar Shahidi, Armin Zavareh, Connor Richards, Lissa Taitano, Kamshad Raiszadeh

**Affiliations:** Department of Orthopaedic Surgery, University of California, San Diego, La Jolla, CA 92093, United States; Department of Orthopaedic Surgery, University of California, San Diego, La Jolla, CA 92093, United States; Livara Health, San Diego, CA 92018, United States; Livara Health, San Diego, CA 92018, United States; Livara Health, San Diego, CA 92018, United States

**Keywords:** Stenosis, spine, pain, conservative, spinal, management

## Abstract

**Importance:**

Spine pain is a prevalent and costly condition affecting up to 85% of individuals throughout their lifetime, and spinal stenosis is one of the most debilitating sources of spine pain. Although conservative management is the first line of treatment for spinal stenosis, radiographically severe cases often are directly referred to surgical intervention due to the belief that conservative strategies delay necessary treatment. However, no studies support the premise that individuals with more radiographically severe stenosis respond poorly to conservative management.

**Objective:**

The purpose of this study was to compare improvements in pain, disability, strength, medication usage, and patient function after an exercise-based physical therapist-led program.

**Design:**

This study was a retrospective analysis of a single arm clinical trial.

**Setting:**

This was in the setting of an outpatient exercise-based physical therapist-led program.

**Participants:**

One thousand eight hundred six individuals with radiographically mild, moderate, or severe lumbar spine stenosis participated in this study.

**Intervention:**

The intervention was an exercise-based physical therapist-led program.

**Outcomes and Measures:**

Outcome measures included pain, low back pain related disability, patient specific function, analgesic medication use, and lumbar extensor strength.

**Results:**

Participants demonstrated significant improvements in all variables of interest, and 11.5% of participants reported cessation of narcotic use with treatment. There were no significant differences in treatment response across radiographically mild, moderate, or severe stenosis groups for any outcome.

**Conclusions:**

Exercise-based rehabilitation is as beneficial in the short term for individuals presenting for nonoperative care with radiographically severe stenosis compared to their milder counterparts.

**Relevance:**

Exercise-based rehabilitation results in the same magnitude of improvement in individuals with chronic low back pain regardless of radiographic severity of spinal stenosis.

## Introduction

Low back pain (LBP) is a highly prevalent musculoskeletal condition that is responsible for the greatest number of years lived with disability in the general population.^1–6^ Between 65% and 85% of individuals experience LBP at some point in their life.^7^ The presence of symptomatic lumbar spinal stenosis has a strong negative influence on health related quality of life.^8^ Of symptomatic spinal pathologies, spinal stenosis is one of the most common, costly, and difficult to remediate. Data on the natural history of stenosis is mixed, with some studies reporting favorable short-term (<1 year) outcomes in 50% of patients,^9^ and others report that up to 60% experience symptom deterioration over time.^10^ Further, longer term outcomes indicate that a significant proportion (between 15% and 30%) of individuals with stenosis experience worsening of stenosis.^11^^,^^12^ Importantly, although radiographic severity of stenosis has not been consistently shown to be associated with clinical severity,^13^ symptomatic individuals with more structurally severe stenosis are more likely to be surgically managed instead of conservatively managed.^14^

Initial conservative management of spinal stenosis is typically non-operative. However, conservative management for stenosis is not standard worldwide, and often moderate and severe cases, often defined according to severity of structural changes, are streamlined to more immediate surgical intervention.^15^^,^^16^ The literature on efficacy of surgical management as compared to conservative management is conflicting; some studies report that surgical management is superior to conservative management,^17–19^ however these studies also report high incidence of side effects (10%-30%) and most have inclusion criteria requiring eligible participants to have already failed an initial trial of 3 to 6 months of conservative management,^20^ potentially biasing results in favor of surgical management. On the other hand, some studies demonstrate that conservative management is equivalent to surgical treatment,^20^^,^^21^ or is even superior in mild cases.^22^ Given these conflicting reports, reviews of the literature evaluating the comparative efficacy of conservative versus surgical management of spinal stenosis state that the quality of the literature is too poor to allow for clear clinical recommendations to be made.^20^

One factor confounding these investigations is that conservative management is broadly defined. Individual or combined applications of pharmacological management, physical therapy, injections, and education are often discussed interchangeably under the umbrella of “conservative treatment” when comparing to surgical management. However, the individual components of these treatments have different physiological targets, dosing regimens, and different treatment effects. Of the common conservative treatments, exercise-based rehabilitation has shown to demonstrate consistent efficacy; with studies reporting that a large proportion of patients with stenosis participating in a physical therapist program with an exercise component experience short and long term symptom improvement (up to 3 years),^23–25^ and have reduced incidence of surgery.^9^ However, some evidence suggests that these improvements are not observed when the level of radiographic stenosis is considered severe.^24^^,^^26^^,^^27^ Indeed, some proposed clinical decision-making algorithms recommend that individuals with severe stenosis should not be considered for physical therapist-based treatment at all and should be prioritized for surgical intervention in order to reduce further disease progression.^15^^,^^16^ To date, very little evidence exists that supports or refutes the differential efficacy of exercise-based rehabilitation programs across individuals with varying levels of radiographic spinal stenosis. Therefore, the purpose of this investigation was to evaluate whether improvements in pain, disability, strength, medication usage, and patient goals in response to an exercise-based rehabilitation program differed across individuals with mild, moderate, or severe radiographic lumbar spine stenosis as defined by imaging studies. Our hypothesis was that individuals with severe radiographic stenosis would demonstrate significantly smaller improvements in outcomes as compared to those with mild or moderate radiographic stenosis.

## Methods

### Participants

This was a secondary analysis of data from a clinical trial registry aimed at understanding the efficacy of exercise-based rehabilitation on individuals with spine pain and structural pathology (NCT #04081896) approved by the local ethical review board (WIRB #1252493). All participants provided informed consent to participate in this study. As part of the secondary analysis, we constrained eligibility to a sub-cohort of individuals with radiographic evidence of lumbar spinal stenosis who participated in a supervised in-clinic exercise-based rehabilitation program. Participants were included if they were (1) prescribed exercise-based rehabilitation to address their spinal condition; (2) had radiographic imaging within 6 months of initiation of their exercise-based rehabilitation program; (3) that imaging indicated stenosis at one or multiple levels in the lumbar spine; and (4) they had complete baseline pain and disability data. Participants were required to have undergone at least 3 treatment sessions to evaluate the influence of the program on patient outcomes, and they must have completed their prescribed program within 6 months of an initial evaluation.

### Exercise-based rehabilitation program

The exercise-based rehabilitation program was administered in an integrated practice unit (IPU) consisting of a multidisciplinary treatment team of physical therapists, orthopedic spine surgeons, spine-trained physician assistants, and pain specialist consultants. The exercise-based rehabilitation treatments were implemented under direct supervision of a licensed physical therapist or physical therapist assistant, and included machine-based resistance exercises prescribed and progressed as previously described in detail^28^ along with directional preference exercises, and patient education on sleep, nutrition, posture, and ergonomics as needed based on impairments identified upon initial evaluation.^29^ The exercise-based rehabilitation program was prescribed based on an initial dose intensity of 60% of a maximum voluntary contraction into extension performed on a lumbar extension isokinetic dynamometer (MedX Holdings Inc., Cheyenne, WI, United States). Machine-based lumbar extension resistance exercises were then performed at this intensity for 15 to 20 repetitions throughout their available range of motions and within symptom tolerance. Exercise intensity (load) was progressed by 5% to 10% in subsequent visits if participants were able to tolerate >20 repetitions without increased pain; remained unchanged if participants tolerated between 10 and 20 repetitions; and was decreased by 5% to 10% if they were unable to perform at least 10 repetitions. Exercise frequency and duration was recommended at 2 times per week for 10 weeks, however flexibility as afforded to participants according to their scheduling capacity and preferences.

### Outcomes measured

The primary predictor variable of interest was radiographically determined stenosis severity, categorized as mild, moderate, or severe based on radiology reports from imaging obtained prior to participation in the exercise-based rehabilitation program. Severity was based on the specific notation from each radiographic report from magnetic resonance images or computed tomography images. Severity grades were dependent on the preferred grading system of the reading radiologist. For individuals with multiple levels of stenosis, they were categorized according to the spinal level with the most severe designation. Primary outcomes included changes in pain, disability, medication use, patient goal achievement, and paraspinal strength. Pain was measured using a visual analog scale from 0 to 100 points and was collected for the back and legs as appropriate.^30^ Because leg pain is a common symptom of lumbar spinal stenosis, and varies by side according to the structural pathology of the stenosis, it was collected bilaterally, the maximum value was used across the left and right leg as the representative leg pain value. A reduction in score of at least 15 points on this scale is considered a clinically meaningful reduction.^31^ Functional status was measured using the Oswestry Disability Index (ODI), which is a validated questionnaire that provides information on low-back pain related disability including features that are commonly limited in individuals with spinal stenosis such as walking, standing, and traveling.^32^ A reduction in ODI score of 10 points or more is considered a clinically important improvement in this measure.^33^ The use of narcotic medications for pain management was categorized according to frequency of use (none, <1/day, 1-2/day, 3-5/day, and >6/day). Patient goals were measured using the Patient Specific Functional Scale (PSFS), which is a 0 to 10 scale indicating the patient’s ability to perform self-entered activities or goals, with 0 indicating the inability to perform an activity and 10 indicating the ability to perform the activity at an uninjured level.^34^ This measure was utilized as it allows for a more personalized characterization of functional limitations as compared to other more generic measures, and allows individuals to identify personally relevant activities (eg, walking, sports, hobbies) that are important to the patient. This measure has been shown to be reliable and valid for measuring function in individuals with lumbar pathology.^35^ An increase in score of at least 1.6 points is considered a minimal clinically important difference for this measure.^36^ Strength of the lumbar paraspinal muscles was measured for resisted extension using a MedX isokinetic dynamometer in order to assess impairment-level changes in core strength as this was a major component of the treatment program. Response to the program was measured as the change in outcomes from baseline to the time of discharge. Covariates included demographic, stenosis-specific, and program-specific variables. Demographic variables included age, sex, and body mass index (BMI). Stenosis-specific variables included stenosis type (central or foraminal). Program-specific variables included the number of days in the program and number of visits attended.

### Statistical analysis

Each measurement was compared across stenosis severity groups using a one-way Analysis of Variance (ANOVA) with Sidak post-hoc corrections for multiple comparisons for continuous variables, and chi-square analysis for categorical or binary variables. Data were confirmed for homogeneity of variance using Levene Test. Both intention-to-treat based analysis and as-treated analysis approaches were used to evaluate outcomes. For intention to treat analyses, any missing outcomes were assumed to have not changed from the baseline. In the as-treated analysis, only complete data was analyzed for a given outcome. Secondary covariates that were significantly different across stenosis severity groups were included in a multivariate linear regression model (for continuous outcomes) or logistic regression model (for categorical outcomes) along with the primary predictor of interest to adjust for confounders. Chi-square or Fisher Exact testing were used to evaluate whether there were differences in proportions of individuals who demonstrated positive and/or clinically meaningful responses (where minimal clinically important difference values were available) to treatment. Adjusted *P* of <.05 were considered statistically significant.

### Role of the funding source

The funders played no role in the design, conduct, or reporting of this study.

## Results

### Participants

**Figure 1:**
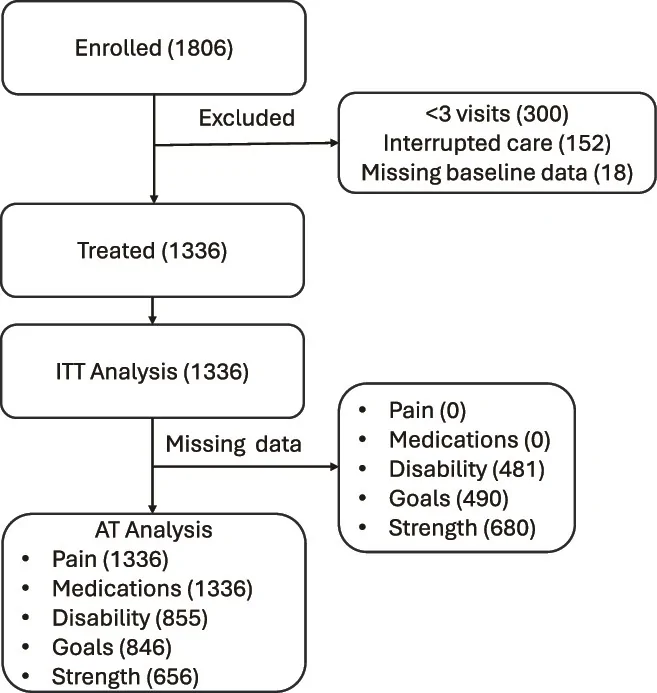
Consort Flow Diagram. Abbreviations: AT = as treated; ITT = intention to treat.

An initial sample of 1806 individuals initiated an exercise-based rehabilitation program for their spine condition. Of those, 300 participants were excluded due to completing less than 3 visits, and 152 participants were excluded due to breaks in care resulting in more than 6 months treatment duration. Another 18 did not have baseline data for the outcomes of interest. To explore whether pragmatic aspects of practice were biasing results, we compared the baseline characteristics and treatment outcomes between the included cohort and those who were excluded due to completing less than 3 visits to ensure their exclusion did not impact interpretation of the results. Those who attended less than 3 visits had a greater BMI, greater prevalence of central stenosis, lower narcotic use, and higher disability, although the magnitude of these differences was not clinically meaningful (Supplementary Material 1). There were 1336 (74%) participants resulting for analysis (Figure 1). Individuals with mild radiographic stenosis represented 19.3% of the cohort, those with moderate radiographic stenosis represented 36.6% of the cohort, and those with severe radiographic stenosis represented 44.1% of the cohort. The majority of participants were in their sixth decade of life, were overweight, and were female individuals with severe radiographic stenosis were significantly older (*P* < .001), heavier (*P* = .04), and male participants were more prevalent (*P* = .02) in those with mild radiographic stenosis. Of the 1336 participants who initiated the program, all participants (100%) had complete pain and medication use outcomes data at the time of discharge, and 64.0% had both pain and disability data upon discharge. Individuals who did not have disability data upon discharge still participated fully in the program but did not complete the disability questionnaire. At baseline, participants reported moderate levels of pain and disability, low-moderate ability to achieve goals, and had chronic (>3 months duration) symptoms. Most of the stenosis was classified as central, with central stenosis being more prevalent in those with severe radiographic stenosis as compared to mild or moderate (*P* < .001). Duration in the program averaged approximately 13 visits over the course of 92 days and ranged from 11 to 182 days with no differences in attendance across radiographic stenosis severity types (*P* > .09). Baseline characteristics across radiographic stenosis severity groups can be found in the Table 1.

**Table 1:** Baseline characteristics of participants^a^

Variable	*N*	Mild	Moderate	Severe	*P*
Age	1336	57.12 (15.07)	64.76 (14.70)	71.19 (11.48)	<.001^b^
BMI	1333	27.05 (5.48)	28.01 (5.79)	28.05 (5.50)	.04^b^
Sex (% female)	1336	53.5%	55.0%	47.0%	.02^b^
Days in program	1336	90.80 (35.44)	91.98 (38.23)	92.39 (36.84)	.85
Number of visits	1336	12.47 (6.62)	13.40 (7.00)	13.58 (6.93)	.09
Symptom duration (d)	1282	73.38 (112.12)	74.68 (105.81)	90.23 (127.26)	.05
Stenosis type (% central)	1336	41.1%	53.4%	65.4%	<.001^b^
ODI initial	1336 (855 at FU)	30.62 (14.59)	32.62 (15.07)	33.16 (14.97)	.07
PSFS initial	1322 (846 at FU)	3.13 (2.08)	3.17 (2.02)	3.14 (2.18)	.95
Pain	1336 (1336 at FU)	57.29 (18.65)	58.58 (20.84)	60.31 (21.23)	.12
Lumbar extensor strength	1069 (656 at FU)	142.53 (80.76)	132.67 (76.29)	137.64 (80.13)	.32
Narcotic medication use (%)	1336 (1336 at FU)				.73
None	1029	20.0%	36.5%	43.4%	
<1/d	111	18.0%	37.8%	44.1%	
1-2/d	110	19.1%	31.8%	49.1%	
3-5/d	74	12.2%	40.5%	47.3%	
>6/d	12	16.7%	50.0%	33.3%	

^a^Values are represented as mean (SD) or percent. Abbreviations: BMI = body mass index; FU = follow up; ODI = Oswestry Disability Index; PSFS = Patient Specific Functional Scale.

^b^Significant *P.*

### Intention to treat analysis

**Figure 2:**
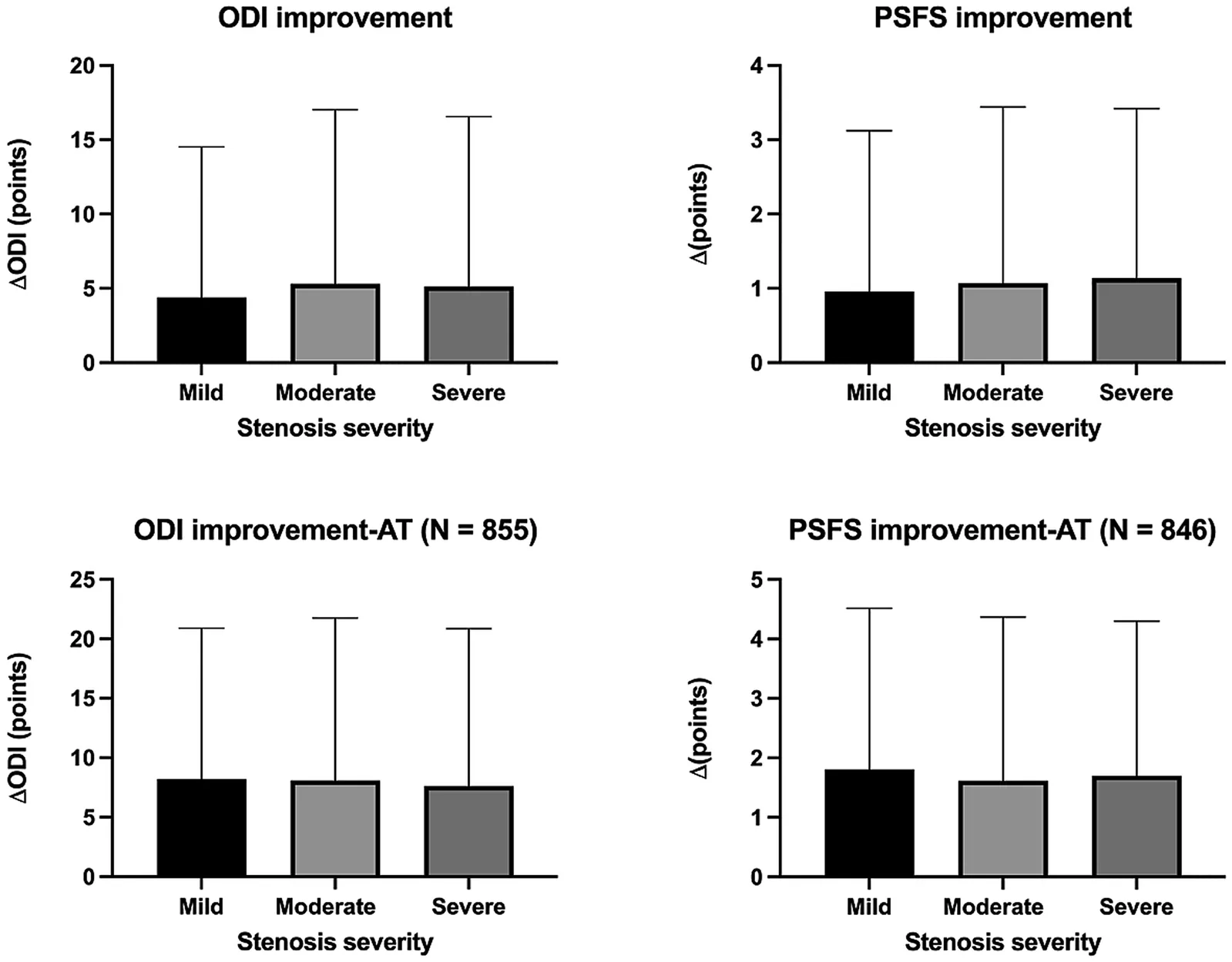
Improvements in Disability and Goal Achievement Scores Across Groups for the Intention to Treat Analysis (Top Row) and the As-Treated Analysis (Bottom Row). Data are represented as unadjusted mean and SD. Abbreviations: AT = as treated; ODI = Oswestry Disability Index; PSFS = Patient Specific Functional Scale.

**Figure 3:**
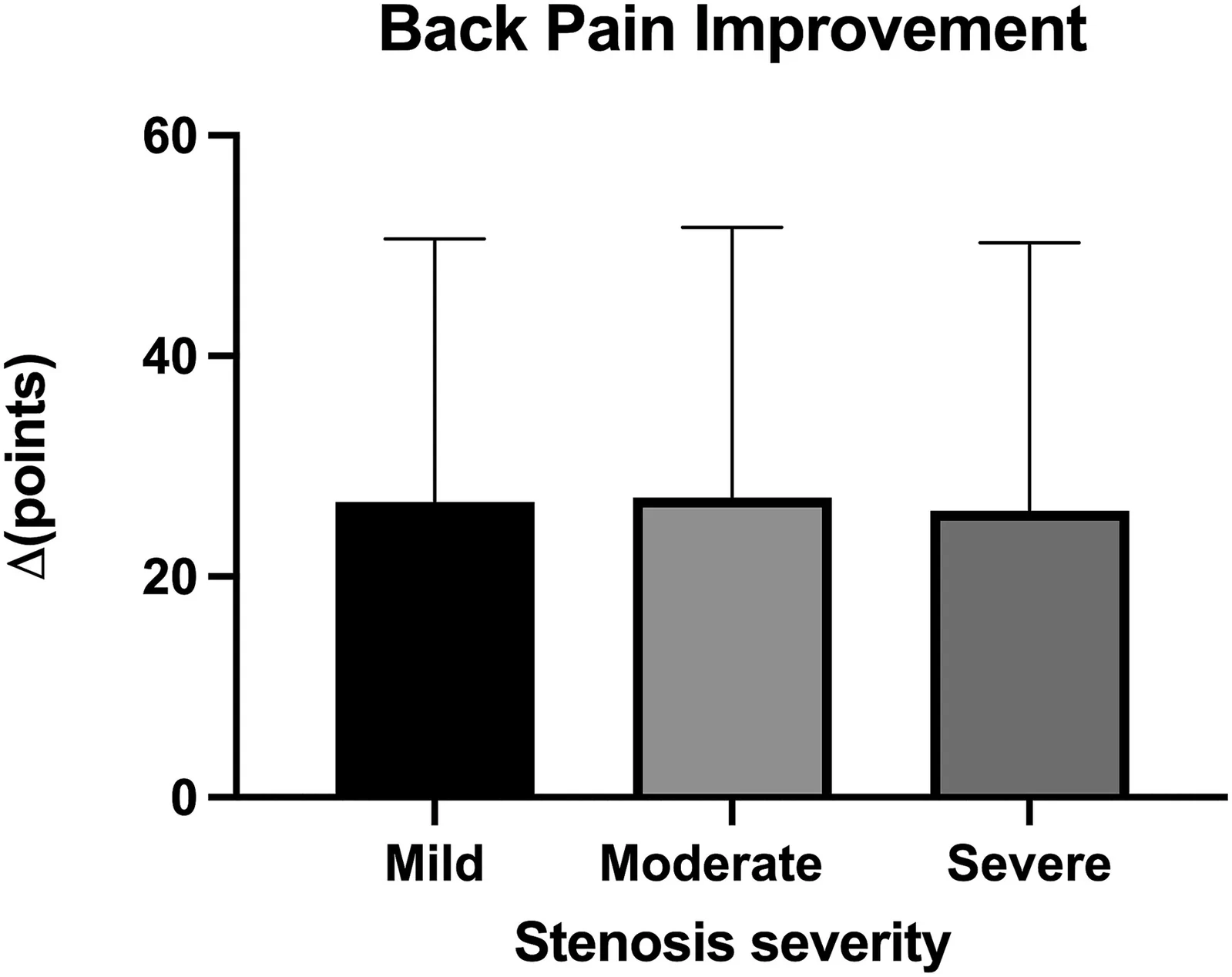
Improvements in Back Pain for All Participants Across Stenosis Severity Groups. Data are represented as unadjusted mean and SD.

**Figure 4:**
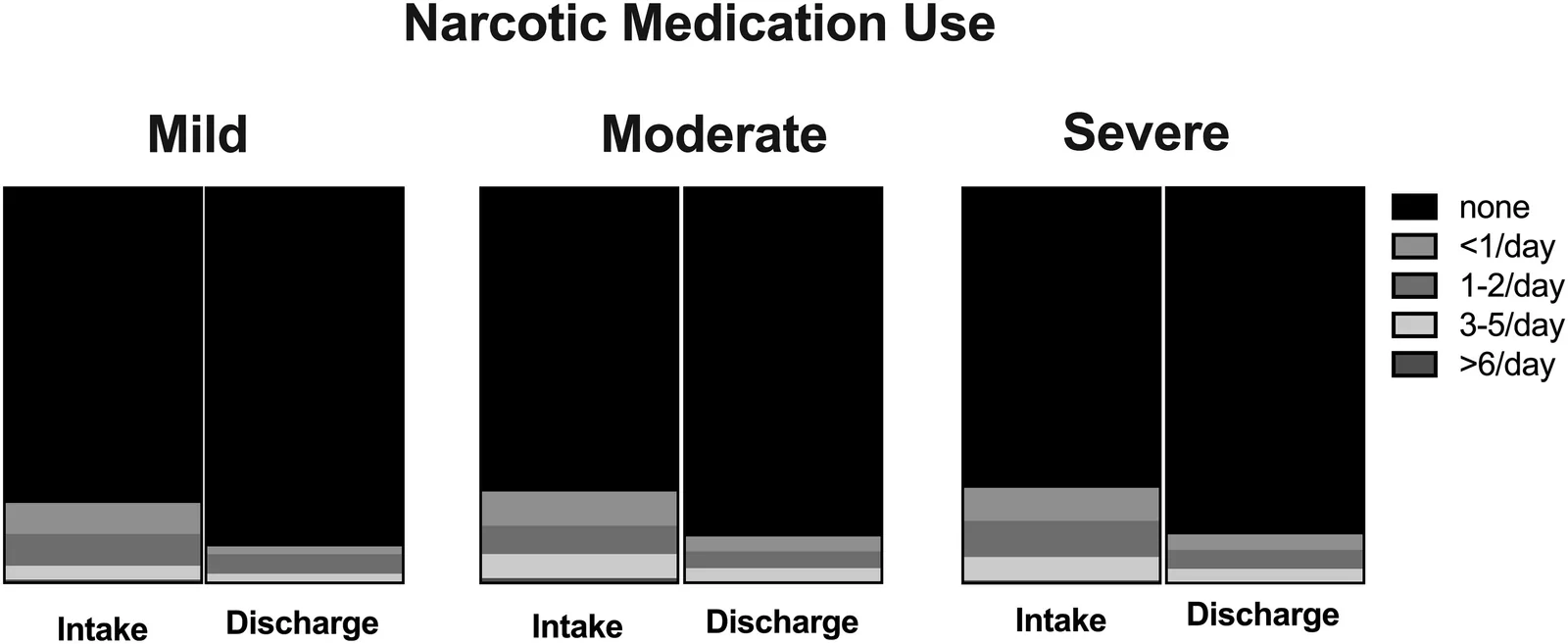
Changes in Frequency of Narcotic Medication Use Across Mild, Moderate, and Severe Stenosis Categories.

**Figure 5:**
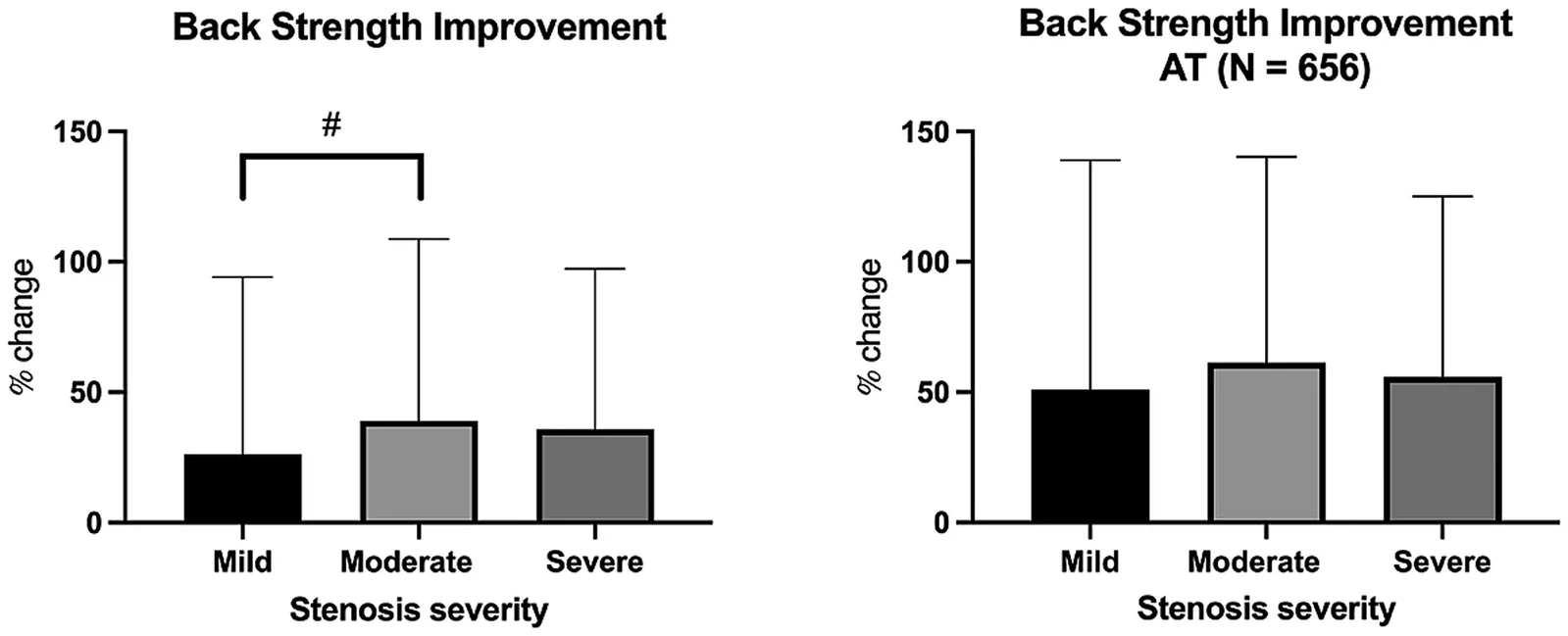
Changes in Back Extensor Strength as Measured by an Isokinetic Dynamometer for the Intention to Treat (Left) Analysis and As-Treated (AT; Right) Analysis. Data are represented as unadjusted mean and SD.

For the intention-to-treat outcomes, the average (SD) improvement in ODI was 5.1 (11.3) points (*P* < .001), with 16% of the cohort achieving at least a 50% improvement in disability. Average improvement in goal achievement (PSFS score) was 1.7 (2.7) points (*P* < .001; Figure 2), with 32.9% achieving at least a 50% improvement in goal achievement. Average improvement in pain was 26.6 (24.3) points (*P* < .001; Figure 3). There were no significant differences in the magnitude of improvement reported for disability, goal achievement, or pain across radiographic stenosis severity groups (*P* > .49). The frequency of narcotics use was reduced in 15.6% of patients, with 11.5% ceasing use of narcotics altogether (Figure 4). There was no significant difference in narcotic use reduction across groups (*P* = .72). Participants improved their lumbar extension strength an average of 35.1 (66.1)% (*P* < .001). Figure 5 illustrates improvements in back extension strength in response to treatment. Adjusting for significant covariates of age, BMI, sex, and stenosis type did not change the results, nor did accounting for baseline levels for each outcome. Similarly, the inclusion of individuals who attended less than 3 visits did not change the results. There were no differences in proportion of responders across radiographic stenosis severity categories for disability or goal achievement (*P* > .60).

### As-treated analysis

Due to lack of complete data for some outcomes, disability, goal achievement, and strength outcomes were also included in an as-treated analysis. Complete data were available for 64.0% of patients for disability, 63.3% of patients for PSFS scores, and 49.1% of patients for strength scores. Individuals who did not have complete follow up data were younger (62.1 [14.9] years old), stronger (144.94 [88.9] ft × lb, *P* = .02), and more symptomatic (ODI 33.6 [15.9], *P* = .02; Pain 60.7 [20.2], *P* = .03) than the cohort with complete data, in addition to having a smaller proportion of individuals with severe radiographic stenosis (47.7% mild, 34.3% moderate, 33.7% severe; *P* < .001). The average improvement in ODI was 7.9 (13.3) points (*P* < .001), with 25% of participants achieving at least a 50% improvement in disability. Average improvement in goal achievement (PSFS score) was 1.7 (2.7) points (*P* < .001), with 51.7% of participants achieving at least a 50% improvement in goal achievement. There were no differences in the magnitude of improvement reported for disability or goal achievement across radiographic stenosis severity groups (*P* > .77; Figure 2). Lumbar paraspinal strength improved by an average of 57.1 (76.5)%, and there were no differences in magnitude of strength improvement across radiographic stenosis severity groups in the as-treated analysis (*P* = .47; Figure 5). There were no differences in proportion of responders across categories for strength (*P* = .07) or disability (*P* = .32).

## Discussion

This study demonstrated that individuals with varying levels of radiographically defined stenosis severity experienced similar, improvements in pain, disability, goal achievement, and spinal extensor strength, along with reductions in narcotic medication use in association with participation in an exercise-based interdisciplinary rehabilitation program. These improvements were clinically meaningful (reached minimal clinically important difference thresholds) for pain, but not disability or goal achievement regardless of the analysis approach (intention to treat vs. as-treated).

Our results are consistent with previous literature demonstrating that interventions including exercise can be beneficial for improving pain and function in individuals with lumbar spine stenosis when compared to no treatment,^23–25^^,^^37^ particularly in the short term. However, the radiographic severity of stenosis and its independent association with treatment response has not been explicitly investigated. More common investigations are those that compare responsiveness across varying symptom severities, and frequently report that individuals with more severe symptoms are more likely to require health care management beyond what is available with conservative management strategies.^38^ Our findings confirm that more severe radiographic stenosis was not associated with greater symptom severity or functional deficit; instead, demographic and structural characteristics such as age, sex, and stenosis type were related. These findings provide important evidence that not only is exercise-based rehabilitation not detrimental to those with more radiographically severe stenosis, it is similarly beneficial across structural presentations. The lack of relationship between structural features and symptom severity is in line with several previous reports acknowledging that changes in spinal anatomy as a function of age and sex are common observations, regardless of the presence of symptoms.^39–41^ Indeed, the prevalence of spinal stenosis in imaging of asymptomatic older individuals, the frequently ambiguous and inconsistent symptoms, and the diverse nature of spinal stenosis all contribute to the complexity of its management.

Despite the improvements observed across all groups in the measured outcomes, many of these improvements did not reach clinically meaningful levels according to defined thresholds.^42^ Their magnitudes are similar to previous studies using similar outcomes for pain and disability^20^^,^^37^ but demonstrated greater reductions in medication use.^23^ Given the observation that a significant proportion of individuals reduced their analgesic medication intake after treatment, it is possible that associations between treatment and symptom reduction are confounded by this concurrent change. The optimal balance between pharmacological pain management and symptom resolution requires further elucidation, particularly considering the high prevalence of narcotic use and misuse in populations with chronic spine pain.^43^

This study is not without limitations. One major limitation is that many clinicians consider lumbar spine stenosis to be primarily defined according to symptoms of claudication, and not primarily defined according to radiographic severity. Given the high prevalence of degenerative findings on imaging studies in asymptomatic individuals, this is an important caveat to the interpretation of the findings of our study. However, individuals from this study were actively seeking treatment related to symptoms presumably associated with these imaging findings, although specific symptoms of claudication were not objectively documented. Another limitation is that our follow-up duration only spanned the duration of the treatment program (approximately 3 months) and as such is likely insufficient for assessing the durability of the observed effects over the long term. Third, our study design was a secondary analysis of a clinical trial, in which we analyzed a sub-group of individuals with radiographic stenosis who were participating in an exercise-based rehabilitation program. Given that this secondary design lacked a control group, it is inherently susceptible to both selection bias and natural history bias of spinal stenosis across different severities over time. However, given that the duration of symptoms was on average 6 months or more in our cohort, the likelihood that large variations in symptoms within the study period are unrelated to treatment are less likely. Our cohort was not balanced across groups according to some baseline characteristics, which may have led to confounding influences of age, sex, and stenosis type (central or foraminal) on interpretation of treatment outcomes. Although we attempted to mitigate this issue by using a multivariate analysis to adjust for these confounders, a more rigorous study design is necessary to address this limitation. Finally, this study may suffer from selection bias in that it is possible that individuals that had more severe symptoms either elected not to initiate this modality of conservative management at all, initiated but did not continue, or were deemed by their referring provider to not be fit for conservative management. Indeed, in our evaluation of those who initiated and did not continue past 1 to 2 visits showed small but significant differences in baseline characteristics, however these differences did not influence the overall findings of the study. Regardless, pragmatic factors influencing the choice to continue treatment after initiation should be considered. Additional selection bias may also exist; however, many insurance providers will not approve surgical interventions until individuals have demonstrated failure in some form of conservative management. Future research endeavors should aim to investigate whether short-term enhancements in pain and functional capacity in individuals with severe stenosis are sustained over an extended duration, and whether exercise-based physical therapy can effectively forestall the necessity of long-term transition to more costly and invasive interventions such as surgery.

## Conclusions

Individuals with severe radiographic stenosis undergoing an exercise-based rehabilitation program demonstrate similar short-term improvements in spine pain, disability, goal achievement, strength, and narcotic use cessation when compared to those with mild or moderate radiographic stenosis who presented for nonoperative treatment to an IPU. Further research is needed to evaluate the durability of these improvements and if they influence the likelihood of progression to more invasive and costly interventions such as surgery in the long term.

## Supplementary Material

PTJ-2026-0149_R1_Supplementary_Material_pzag084

## Data Availability

Data will be made available upon reasonable request to the corresponding author.
