# Supplementary material for: Severity of structural lumbar spinal stenosis does not impact responsiveness to exercise-based rehabilitation
Source: Phys Ther. 2026 Aug 8;106(9):pzag084. doi: 10.1093/ptj/pzag084 (PMC13613343; doi:10.1093/ptj/pzag084)
Supplement: Supplementary file 1 [file ptj-2026-0149_r1_supplementary_material_pzag084.pdf]

Supplementary Material.

Variables	<3 visits (N=300)	Included Cohort	p-value
Age (years)	65.2(14)	66.12(14)	0.68
BMI (kg/m2)	28.7 (8.2)	27.8(5.6)	0.02
Symptom duration (months)	94 (123)	81.2(117)	0.09
Sex(F:M)	168:129	684:652	0.09
Stenosis (Central:Foraminal)	160:140	584:752	0.003
Narcotics (None:<1/day:Daily)	210:27:60	571:257:508	<0.001
ODI (points)	37 (17)	33 (15)	<0.001
PSFS (points)	3.2(2.3)	3.2(2.1)	1.00
Pain (points)	60.7(20.1)	59.1(21)	0.23
Strength (ft*lbs)	135.7(83)	136.9(78.9)	0.82
DeltaODI (points)	N/A	7.9(13.3) AT; 5.1(11.3) ITT	-
DeltaPSFS (points)	N/A	1.7(2.7)	-
DeltaPain (points;N=105)	5.6(15.9)	26.6(24.3)	<0.001
DeltaStrength (% change)	N/A	57.1(76.5%) AT; 35.1(66.1%) ITT	-

Values are presented as mean(SD) or N.

BMI: Body Mass Index; F: Female; M: Male; ODI: Oswestry Disability Index; PSFS: Patient Specific Functional Scale; AT: As-treated; ITT: Intention to Treat
